# Acceptability, Feasibility and Preliminary Evaluation of a Novel, Personalised, Home-Based Physical Activity Intervention for Chronic Heart Failure (Active-at-Home-HF): a Pilot Study

**DOI:** 10.1186/s40798-019-0216-x

**Published:** 2019-11-27

**Authors:** Nduka C. Okwose, Leah Avery, Nicola O’Brien, Sophie Cassidy, Sarah J. Charman, Kristian Bailey, Lazar Velicki, Iacopo Olivotto, Paul Brennan, Guy A. MacGowan, Djordje G. Jakovljevic

**Affiliations:** 1 Cardiovascular Research Theme, Translational and Clinical Research Institute, Faculty of Medical Sciences, Newcastle University, William Leech Building M4.074, Newcastle upon Tyne, NE2 4HH UK; 2 Centre for Rehabilitation, Exercise and Sports Science, School of Health & Social Care, Teesside University, Tees Valley, UK; 3 Department of Psychology, Northumbria University, Newcastle upon Tyne, UK; 4 Newcastle upon Tyne Hospitals, NHS Foundation Trust, Newcastle upon Tyne, UK; 5 Faculty of Medicine, University of Novi Sad, Novi Sad, Serbia; 6 Department of Cardiovascular Surgery, Institute of Cardiovascular Diseases Vojvodina, Sremska Kamenica, Serbia; 7 Cardiomyopathy Unit and Genetic Unit, Careggi University Hospital, Florence, Italy; 8 Institute of Genetic Medicine, Faculty of Medical Sciences, Newcastle University, Newcastle upon Tyne, UK; 9 RCUK Centre for Ageing and Vitality, Newcastle University, Newcastle upon Tyne, UK

**Keywords:** Physical activity, Chronic heart failure, Home-based intervention, Behavioural change

## Abstract

**Purpose:**

Less than 10% of heart failure patients in the UK participate in cardiac rehabilitation programmes. The present pilot study evaluated feasibility, acceptability and physiological effects of a novel, personalised, home-based physical activity intervention in chronic heart failure.

**Methods:**

Twenty patients (68 ± 7 years old, 20% females) with stable chronic heart failure due to reduced left ventricular ejection fraction (31 ± 8 %) participated in a single-group, pilot study assessing the feasibility and acceptability of a 12-week personalised home-based physical activity intervention aiming to increase daily number of steps by 2000 from baseline (Active-at-Home-HF). Patients completed cardiopulmonary exercise testing with non-invasive gas exchange and haemodynamic measurements and quality of life questionnaire pre- and post-intervention. Patients were supported weekly via telephone and average weekly step count data collected using pedometers.

**Results:**

Forty-three patients were screened and 20 recruited into the study. Seventeen patients (85%) completed the intervention, and 15 (75%) achieved the target step count. Average step count per day increased significantly from baseline to 3 weeks by 2546 (5108 ± 3064 to 7654 ± 3849, *P* = 0.03, *n* = 17) and was maintained until week 12 (9022 ± 3942). Following completion of the intervention, no adverse events were recorded and quality of life improved by 4 points (26 ± 18 vs. 22 ± 19). Peak exercise stroke volume increased by 19% (127 ± 34 vs. 151 ± 34 m/beat, *P* = 0.05), while cardiac index increased by 12% (6.8 ± 1.5 vs. 7.6 ± 2.0 L/min/m^2^, *P* = 0.19). Workload and oxygen consumption at anaerobic threshold also increased by 16% (49 ± 16 vs. 59 ± 14 watts, *P* = 0.01) and 10% (11.5 ± 2.9 vs. 12.8 ± 2.2 ml/kg/min, *P* = 0.39).

**Conclusion:**

The Active-at-Home-HF intervention is feasible, acceptable and effective for increasing physical activity in CHF. It may lead to improvements in quality of life, exercise tolerance and haemodynamic function.

**Trial Registration:**

www.clinicaltrials.gov NCT0367727. Retrospectively registered on 17 September 2018.

## Key Points

- A personalized, home-based physical activity intervention is acceptable and feasible and could lead to improvement in exercise tolerance and quality of life in chronic heart failure patients.
- Increasing step count by at least 2000 steps per day may be a realistic goal for chronic heart failure patients.

## Background

The benefits of cardiac rehabilitation in chronic heart failure (CHF) have been well documented [1]. Evidence-based clinical guidelines recommend that physical activity is integrated into cardiac rehabilitation as a cornerstone of clinical management of CHF [2]. Meta-analyses have demonstrated that increased physical activity can improve functional capacity and quality of life, can reduce symptom burden and likelihood of hospitalisation and can improve cardiac function [3, 4]. Consequently, current guidelines now emphasize physical activity as an important component of cardiac rehabilitation in addition to patient education, psychological support and drug therapy [2, 5, 6].

Despite numerous benefits, participation of heart failure patients in cardiac rehabilitation is low. In the United Kingdom (UK), less than 10% of patients with CHF participate in cardiac rehabilitation [7]. Potential explanations include exclusion of cardiac rehabilitation programmes from local commissioning agreements due to limited funds, lack of capacity for supervised programmes and inadequate social support for patients [8]. Other patient factors include unwillingness to participate in cardiac rehabilitation due to difficulties in attending hospitals, work or domestic commitments and reluctance to attend group-based classes [9].

The barriers highlighted above could be potentially overcome by promoting increased physical activity at home. Daily habitual physical activity (i.e. number of steps and active energy expenditure) is inversely related to patients’ symptoms [10]. Walking is an independent predictor of outcomes in patients with advanced CHF [11]. Current physical activity recommendations for adults are 150 to 300 min per week of moderate-intensity activity, or 75 to 150 min per week of vigorous-intensity aerobic physical activity, or an equivalent combination of moderate- and vigorous-intensity aerobic activity while incorporating muscle-strengthening activities if tolerated [6]. For adults who are unable to meet these guidelines due to chronic conditions or disabilities, regular physical activity according to their ability is recommended [6]. As such, it may be challenging to identify a standardised daily physical activity routine appropriate for all heart failure patients’ accounting for individual differences. Pedometer-based interventions have demonstrated that increasing steps per day by approximately 2000–2500 steps leads to improvements in blood pressure [12, 13] and insulin sensitivity [14]. Furthermore, a large cohort study involving 9306 participants reported a 10% risk reduction of cardiovascular events in individuals at high risk of developing type 2 diabetes for every 2000 steps per day increment in daily physical activity observed [15]. Considering these findings, we developed a novel, personalised, home-based (Active-at-Home-HF) physical activity intervention aiming to increase daily physical activity by 2000 steps in patients with CHF. The aim of the present pilot study was to assess feasibility, acceptability and preliminary efficacy of the Active-at-Home-HF intervention.

## Methods

### Study Design

A single-group, pilot study assessed the feasibility, acceptability and preliminary efficacy of a home-based physical activity intervention in adults with CHF with reduced left ventricular ejection fraction. Eligible participants attended the Clinical Research Facility of the Royal Victoria Infirmary, Newcastle upon Tyne, UK, for two separate visits (i.e. before and after the 12-week intervention). Participants were contacted via email, telephone or spoken to in person to discuss the study and given an opportunity to ask questions to ensure they understood the procedure.

### Participants

Potentially eligible patients were identified by cardiologists via medical records from heart failure clinics at the Royal Victoria Infirmary and Freeman Hospital in Newcastle upon Tyne. These patients were subsequently screened by the same cardiologists using the study eligibility criteria. Once eligibility was confirmed, patients were recruited by a member of the research team (NO, SC) by telephone contact. The study included patients with a left ventricular ejection fraction ≤ 40%, diagnosed for at least 3 months, classified according to the New York Heart Association (NYHA) class II–III, clinically stable and receiving an optimal medical treatment. Patients were required to have no contraindications to physical activity and had to be capable of performing activities of daily living independently. Patients were excluded during screening or contact if they had uncontrolled cardiac arrhythmias, myocardial infarction, percutaneous coronary intervention and/or bypass graft surgery up to 3 months previously, severe obesity (i.e. body mass index > 40) and implantation with left ventricular assist device; were currently participating in a cardiac rehabilitation programme; if they already met physical activity recommendations [6]; or were unable to provide informed written consent.

### Clinical Assessments

During baseline and 12-week follow-up visits, patients underwent clinical assessments including quality of life using the Minnesota Living with Heart Failure questionnaire, blood sampling for N-Terminal pro b-type Natriuretic Peptide (NTproBNP) and cardiopulmonary pulmonary exercise stress testing on a semi-recumbent cycle ergometer (Corival, Lode & Groningen, Netherlands) coupled with non-invasive haemodynamic monitoring (NICOM®, Cheetah Medical, Delaware, USA). A graded exercise test protocol was used for cardiopulmonary exercise testing. This involved maintaining a pedal frequency of 60–70 revolutions per minute with workload increasing at the rate of 10 W per minute. The test was terminated when maximal exertion was achieved, or when the patient was unable to maintain the required cycling cadence, or if the patient desired to stop. Physical activity (step count) was measured continuously using a pedometer (Omron Health care, Model no: HJ-321-E, Japan). Patients recorded daily step counts at the end of each day using a paper-based activity tracker, and results were communicated weekly to a member of the study team.

### The Home-Based Physical Activity Programme (Active-at-Home-HF)

The Active-at-Home-HF intervention was designed for patients with CHF to encourage an increase in their overall daily physical activity levels by at least 2000 steps per day from baseline. This behavioural intervention was delivered by telephone to participants in the north-east of England who were patients at the Royal Victoria Infirmary or Freeman Hospital, Newcastle upon Tyne. The intervention team comprised of cardiologists, exercise physiologists and health psychologists. Team members (NCO and SC) involved in monitoring patients were experienced clinical exercise physiologists certified by the American College of Sports Medicine. They also received training delivered by a chartered health psychologist with expertise in health behaviour change (LA) who was also a member of the research team, on the delivery/use of evidence-based behaviour change techniques selected to target physical activity behaviour (e.g., physical activity goal setting, problem solving, self-monitoring) [16, 17]. The same health psychologist developed the brief behavioural intervention, intended for delivery by telephone using a proforma to prompt use of the specific behaviour change techniques. This proforma also served as a record for future discussions following completion during each intervention session delivered. The intervention differed from centre-based programmes in that it focussed on free-living physical activity, did not rely on exercise equipment, was delivered by weekly telephone sessions and focussed on providing participants with the knowledge and behavioural skills to increase and maintain physical activity levels despite the barriers they might face. Once patients enrolled in to the study, they were supported by weekly telephone calls lasting approximately 10 min in duration, designed to initiate, increase and maintain their activity levels. This was achieved through behavioural goal setting where the patient would set a physical activity goal with the guidance and support of a trained research team member. Barriers to reaching the goal were discussed followed by mutual identification of solutions to overcome those barriers. Patients were encouraged to consider times in the past where they had been more physically active as a means of increasing confidence and motivation. Self-monitoring was used to encourage maintenance of activity levels, and patients were prompted to involve family members and friends in their attempts to increase physical activity levels as a means of social support. At the end of each day, the goal was to achieve at least 2000 steps more than the average daily number of steps obtained at baseline as indicated on the pedometer. Physical activity levels were adjusted on an individual basis as conditioning took place, with the emphasis on volume of activity, i.e. duration and number of steps rather than intensity.

### Outcomes

The primary outcomes of interest were acceptability and feasibility of the intervention. Secondary outcomes were changes in functional capacity assessed by peak exercise oxygen consumption and power output, quality of life, haemodynamic function and changes in NTproBNP. Feasibility was defined as willingness of patients to enrol on to the Active-at-Home-HF intervention and was confirmed by recruiting the targeted number of patients. The recruitment target deadline was set at 9 months after recruiting first patient. Acceptability was defined as willingness to engage with and adhere to the intervention and was reported as the percentage of patients who completed intervention. The intervention was considered acceptable if ≥ 80% of patients completed it. This included weekly engagement by telephone and completion of daily physical activity records. If engagement with each of these components was recorded, the intervention was considered acceptable.

### Statistical Analysis

The primary aim of the present study was to assess acceptability and feasibility of the intervention. It is generally accepted that pilot feasibility studies do not require a formal power calculation [18]. However, it was important to assess whether the Active-at-Home-HF intervention, if acceptable and feasible, was capable of improving outcomes of interest to allow a judgement to be made as to whether the intervention is comparable to a centre-based intervention. It was therefore estimated that a sample size of 20 patients would provide sufficient power to detect a clinically acceptable change/increase in peak oxygen consumption of 3 ml/min/kg post-intervention, at the significance level of 5% (*β* = 0.82, *α* = 0.05). The relationship between physical activity and physiological variables was assessed using Pearson’s coefficient of correlation. Statistical significance was indicated if *P* < 0.05. All statistical analyses were carried out using SPSS version 24.0 (SPSS, Chicago, IL, USA).

## Results

### Acceptability and Feasibility

**Fig. 1:**
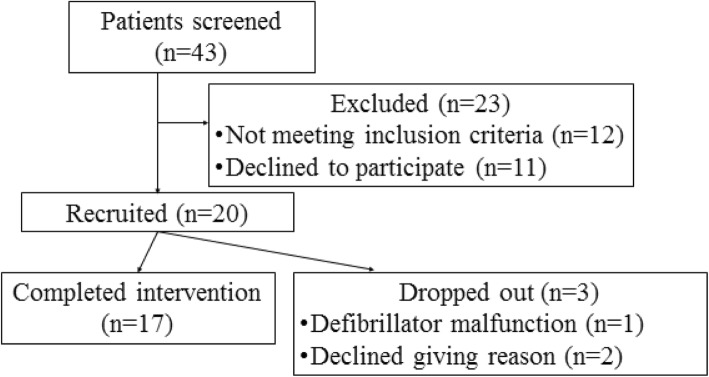
Flow diagram showing patient screening and recruitment into Active-at-home HF intervention

Out of 43 CHF patients contacted by telephone after initial screening, 20 patients met the study inclusion criteria and were willing to take part and were subsequently recruited. Recruitment took place between December 2015 and September 2016. Patients were excluded (*n* = 23) if they already met recommended physical activity guidelines [5] (*n* = 4), were too ill to participate (NYHA stage IV) or were recently hospitalised (*n* = 8). Patients were also excluded if they refused to participate for personal reasons (*n* = 4), time commitment (*n* = 3), ‘feeling not be able to due to age’ (*n* = 1) or being too nervous to participate in a physical activity intervention (*n* = 3). Recruited patients’ demographic and clinical characteristics are presented in Table 1. No adverse events occurred as a result of participating in the intervention/study. Seventeen participants completed the 12-week physical activity intervention. However, two patients were unable to meet or sustain the required minimum target of 2000 steps above baseline due to severe arthritis. The intervention was considered acceptable and feasible as the required number of patients were recruited, and the majority of patients completed the intervention (completion rate 85%; *n* = 17) (see Fig. 1).

**Table 1:** Mean and SD (±) of patients’ demographic and clinical characteristics

Parameter	
Age (years)	68 ± 7
Men/women	18/2
Weight (kg)	84 ± 15
Height (cm)	1.72 ± 0.1
Aetiology of HF (IHD/DCM)	10/10
LVEF (%)	31 ± 8
Medication	
ACE inhibitors	15
β-blockers	20
ARBs	5
Diuretics	13
Anti-arrhythmic	3
NSAIDs/pain killers	6
Warfarin	5
ICD/pacemakers	13
Comorbidities	
COPD	1
Type 2 diabetes	5
Obesity	6
Hypertension	20
Depression	2
Arthritis	1

*ACE* angiotensin converting enzyme, *ARB* angiotensin receptor blockers, *LVEF* left ventricular ejection fraction, *NSAID* non-steroid anti-inflammatory drugs, *IHD*, ischaemic heart disease, *DCM* dilated cardiomyopathy, *COPD* chronic obstructive pulmonary disease, *ICD* implantable cardioverter defibrillator

In situations where patients expressed concerns about their arrhythmias or ischaemia, they were further assessed by the team’s consultant cardiologist and were reassured about safety of participation in the intervention before they took part in the study.

**Fig. 2:**
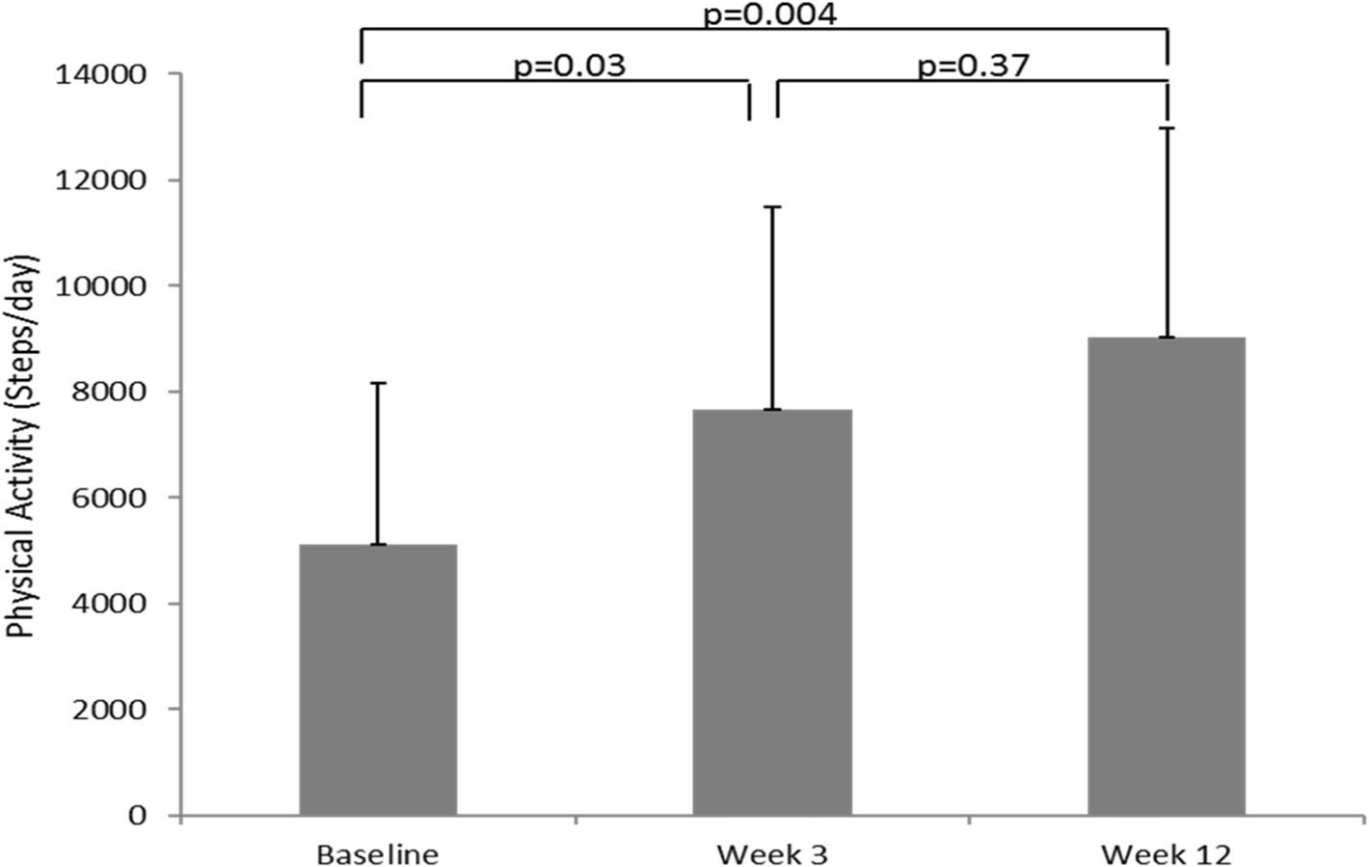
Mean (+ SD) number of steps achieved at baseline and at the end of weeks 3 and 12 of physical activity intervention

The target step count goal of 2000 steps from baseline was achieved at week three with average number of steps per day increasing significantly by 2546 (from 5108 ± 3064 to 7654 ± 3849 steps/day, *p* = 0.03), and was maintained until week 12 (8890 ± 3713 steps/day, Fig. 2). Two patients dropped out of the study for undisclosed reasons, and one participant discontinued due to implantable cardiac defibrillator malfunction.

### Metabolic Changes

There was no statistically significant change in exercise tolerance with peak oxygen consumption and peak workload increasing post-intervention by 4.8% and 11% respectively. However, workload and oxygen consumption at submaximal exercise (i.e. anaerobic threshold) increased by 20% (49 ± 16 vs. 59 ± 14 watts, *P* = 0.01) and 11% (11.5 ± 2.9 vs. 12.8 ± 2.2 ml/kg/min, *P* = 0.39) post-intervention (Table 2).

**Table 2:** Cardio-metabolic changes (mean ± SD) following 12 weeks of Active-at-Home-HF intervention

	Pre-intervention	Post-intervention	*P* value	% change
Measurements at rest				
Oxygen consumption (ml/kg/min)	3.8 ± 1.0	4.1 ± 0.8	0.36	7.9
Respiratory exchange ratio	0.85 ± 0.1	0.85 ± 0.1	0.92	0
Heart rate (beats/min)	67 ± 7	70 ± 7	0.24	4.5
Stroke volume index (ml/beat)	48 ± 9	49 ± 8	0.75	2.0
Cardiac output (l/min)	6.1 ± 1	6.6 ± 1	0.14	8.2
Systolic blood pressure (mmHg)	118 ± 18	124 ± 18	0.41	4.0
Diastolic blood pressure (mmHg)	74 ± 8	76 ± 12	0.74	2.6
Mean arterial pressure (mmHg)	90 ± 9	92 ± 13	0.66	2.2
Measurements at peak exercise				
Oxygen consumption (ml/kg/min)	16.8 ± 3.8	17.6 ± 4.2	0.54	4.8
Respiratory exchange ratio	1.05 ± 0.1	1.07 ± 0.1	0.62	1.9
Heart rate (beats/min)	106 ± 19	107 ± 16	0.92	1.0
Stroke volume (ml/beat)	127 ± 34	151 ± 34	0.05	18.9
Stroke volume index (ml/beat/m^2^)	64 ± 14	75 ± 17	0.04	17.2
Cardiac output (l/min)	13.4 ± 4	15.3 ± 4.9	0.19	14.2
Cardiac index (l/min/m^2^)	6.8 ± 1.5	7.6 ± 2.0	0.19	11.7
Systolic blood pressure (mmHg)	155 ± 30	150 ± 30	0.62	3.2
Diastolic blood pressure (mmHg)	80 ± 8	79 ± 8	0.72	1.3
Mean arterial pressure (mmHg)	103 ± 13	102 ± 13	0.87	1.0
Peak exercise workload (watts)	82 ± 10	91 ± 19	0.21	11
Exercise workload at anaerobic threshold (watts)	49 ± 16	59 ± 14	0.01	20
Oxygen consumption at anaerobic threshold (ml/kg/min)	11.5 ± 2.9	12.8 ± 2.2	0.39	11.3
Rate of perceived exertion	16 ± 2.4	17 ± 2.3	0.22	6.3

### Haemodynamic Changes

The completion of the intervention resulted in significant improvements in peak exercise stroke volume (126.5 ± 33.8 vs. 150.8 ± 33.5 ml/beat, *P* = 0.05) and stroke volume index (64.6 ± 14 vs. 75.2 ± 17 ml/beat/m^2^, *P* = 0.04). There was also a 10–15% improvement in peak exercise cardiac output and cardiac index, although these were not statistically significant (Table 2).

### Blood Biomarkers and Quality of Life

There were no statistically significant changes in metabolic biomarkers following completion of the intervention. There was a 4-point improvement in quality of life score (Table 3).

**Table 3:** Blood biomarkers and quality of life (mean ± SD) following 12 weeks of Active-at-Home-HF

	Pre-intervention	Post-intervention	*P* value	% change
Cholesterol (mmol/l)	4.0 ± 0.9	3.9 ± 0.9	0.59	2.5
Triglyceride (mmol/l)	1.5 ± 0.7	1.8 ± 0.9	0.45	20
HDL (mmol/l)	1.2 ± 0.3	1.1 ± 0.3	0.67	8.3
LDL (mmol/l)	2.1 ± 0.7	1.9 ± 0.7	0.46	9.5
HbA1c (mmol/mol)	49.2 ± 17.3	47.5 ± 12	0.77	3.5
FBG (mmol/l)	6.2 ± 2.9	7.0 ± 3.8	0.56	12.9
NT proBNP (pg/ml)	823 ± 1085	876 ± 1114	0.89	6.4
Renal function eGFR	65.4 ± 18.6	61.4 ± 17.4	0.61	6.1
QoL	26 ± 18	22 ± 23	0.50	15.4

*HDL* high-density lipoprotein, *LDL* low-density lipoprotein, *HBA1c* glycated haemoglobin, *FBG* fasting blood glucose, *NT* proBNP N-terminal brain natriuretic peptide, *QoL* quality of life, *eGFR* glomerular filtration rate

**Fig. 3:**
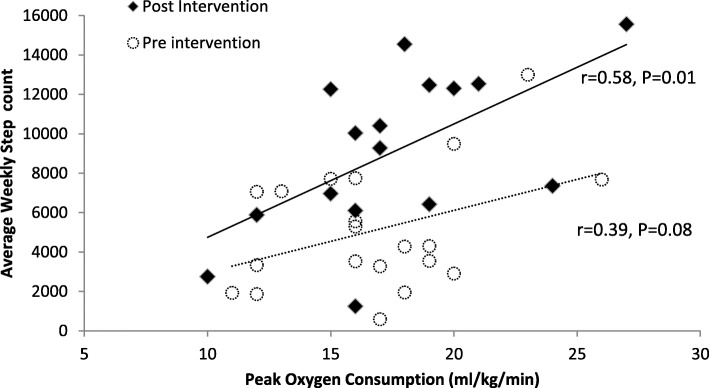
Relationship between number of steps and peak oxygen consumption pre- and post-intervention

Daily number of steps correlated positively with peak oxygen consumption post-intervention (*r* = 0.58, *P* = 0.01), but not pre-intervention (*r* = 0.39, *P* = 0.08). The significant correlation observed post-intervention, although moderate, suggests that daily physical activity is positively associated with functional capacity (exercise tolerance) in active but not sedentary patients with chronic HF (Fig. 3). This suggests that increasing daily walking improves fitness levels in heart failure patients.

## Discussion

The major findings of this study are that the Active-at-Home-HF intervention demonstrated to be both acceptable and feasible to patients with CHF and led to modest changes in exercise tolerance and haemodynamics. This was shown by the number of patients (85%) who engaged with and completed the intervention after enrolment. This figure is comparable to adherence rates (> 75%) reported from centre-based studies [19, 20]. Piotrowicz et al. [20], who compared a home-based tele-monitored cardiac rehabilitation to centre-based rehabilitation, reported 100% adherence in the home-based group. Seventy-five percent of dropouts in the centre-based group of the above study were due to inadequate funds and availability of transport while 25% dropped out due to difficulty in matching the centre-based training with their daily activities. These findings are important because the intervention has shown to provide a viable and potentially low-cost (i.e. brief behavioural intervention delivered by telephone, no reliance on exercise equipment provision in clinical settings) alternative to centre-based programmes for patients not wanting to engage in group-based rehabilitation and is a solution to overcome other barriers including travel constraints.

Patients with heart failure often experience a decline in health-related quality of life. Following clinical presentation/diagnosis, psychological distress can limit activity and lead to a decline in quality of life [21]. Exercise training has been reported to improve quality of life in CHF [22], and a reduction of 5 points or more in the Minnesota living with heart failure questionnaire is accepted as being clinically significant [23]. The present pilot feasibility study reported a 15% (4 point) reduction in quality of life score (i.e. an improvement in quality of life) which is deemed clinically insignificant and as thus contrast studies that have reported a significant improvement in quality of life following exercise intervention [24]. However, a 15% improvement is positive in a group of participants who reported a higher than average quality of life score at baseline (i.e. mean values of 26 points out of a possible score of 105 points in the questionnaire). Cowie et al. [25] also reported no significant change in quality of life in CHF patients following home-based or hospital-based intervention, even though there was a significant improvement in exercise capacity. They further suggested that in older CHF patients, maintaining quality of life rather than improving it might be a realistic aim for a physical activity or rehabilitation programme.

At anaerobic threshold, participants were able to tolerate significantly greater workload (17% increase). This finding is similar to previous studies that also reported a delay in reaching anaerobic threshold [26] and a significant increase in power output at anaerobic threshold [27].

Similarly, increased daily physical activity resulted in a 19% significant increase in peak stroke volume. Our results suggest positive adaptation to the intervention and the improvement of systemic oxygen delivery. Other studies have also reported significant improvements in an echocardiogram-generated stroke volume as a result of long-term (> 12 weeks) exercise training [28]. The capacity to increase physical activity depends on the ability of the heart to generate adequate cardiac output and the ability of skeletal muscles to utilise the oxygen delivered [29]. Therefore, this provides strong evidence for the assessment of cardiac heamodynamics in response to a physical activity intervention. These findings have been extended by other studies which demonstrated that aerobic training also improves diastolic filling, myocardial contractility and left ventricular ejection fraction in individuals with severe left ventricular systolic dysfunction [30, 31]. Based on the findings of the current study and evidence from the above-mentioned studies, it is reasonable to suggest that physical activity in CHF can improve cardiac contractility and stroke volume, potentially leading to reverse remodelling. The present study also found that increased physical activity as recorded from step counts following the Active-at-Home-HF intervention had a stronger correlation with peak oxygen consumption compared with lower step counts (sedentary behaviour) pre-intervention. Jehn et al., who reported a positive correlation between the times spent in light activity/exercise (≤ 3 METs) and improved peak oxygen consumption [11], had demonstrated this previously.

Although the use of steps per day has been criticised for not taking into account exercise intensity [32], a recent study by Tudor-Locke et al. [33] has shown that linear trends for steps per day were statistically significant for cardio-metabolic risk factors including blood pressure for men, weight, waist circumference, insulin, high-density lipoprotein, triglycerides and homeostasis model assessment-estimated insulin resistance. Even at step counts of around 70 steps per minute, which is below the recommended 100 steps per minute suggesting moderate intensity [32], clinically favourable values for many of the cardio-metabolic outcomes were observed. The present study provides further justification for the use of steps per day recommendations in national physical activity guidelines [34]. Furthermore, the present study results are of clinical importance for the management of CHF patients, particularly older adults as most patients have concomitant exercise-limiting co-morbidities such as neuromuscular or orthopaedic problems making the traditional 10,000 step target [35] or steps per minute unrealistic and potentially harmful. As such, it may be appropriate to encourage such patients to exercise at a lower intensity than has been considered necessary to increase maximal exercise capacity.

### Limitations

The following limitations should be considered in the present study. Firstly, it could be argued that sample size limits generalisability of findings. Of the 43 patients screened, only 20 were enrolled into the study meaning a recruitment rate of 46%. However, this was a pilot study with the primary aim of establishing acceptability and feasibility of the intervention. A further criticism could be that this study lacked a control group. However, the primary intention of this study was not to establish the effect of the intervention, instead, it was to assess feasibility and acceptability with a view to informing a larger-scale evaluation (i.e. controlled trial) should the intervention prove to be feasible and acceptable. Secondly, it could be argued that stroke volume was measured non-invasively which is not the gold standard. However, the reproducibility of stroke volume measurements using the NICOM has previously been reported [36]. Lastly, only two female patients were recruited into the study limiting generalisability of the study findings in terms of gender. The nature of this pilot study did not necessitate the use of digital technologies. However, digital technologies which offer additional evaluation of haemodynamic function such as heart rate and blood pressure will be useful in future studies to improve safety of patients while engaging in physical activity, and would likely increase feasibility. Further evaluation of the ACTIVE-at-HOME intervention, clinical and cost-effectiveness is warranted in an adequately powered randomised controlled trial

## Conclusion

The present study demonstrates that the novel, home-based physical activity intervention (i.e. Active-at-Home-HF) is acceptable and feasible and can provide clinical and physiological benefits to people living with CHF. The intervention is associated with increased habitual physical activity level, functional capacity and haemodynamic response to exercise. Significant changes in response to the Active-at-Home-HF intervention were observed in submaximal exercise capacity and cardiac response to exercise. The Active-at-Home-HF intervention provides a viable alternative to centre-based programmes. This helps to overcome barriers including travel cost and reluctance to participate in group-based activity. A larger multicentre study is warranted to further substantiate preliminary findings from the present study.

## Data Availability

The datasets generated during and/or analyzed during the current study are available from the corresponding author on reasonable request.

## Abbreviations

CHF: Chronic heart failure
COPD: Chronic obstructive pulmonary disease
HF: Heart failure
NTproBNP: n-Terminal pro b-type Natriuretic Peptide
NYHA: New York Heart Association
UK: United Kingdom
